# Multiple Signaling Axes (TNF-α/IL1β/IL8, TLR4/MYD88/NF-κB, and TGF-β1/ROS) Associated With Coronary Collateral Circulation of Coronary Chronic Total Occlusion in Geriatric Patients

**DOI:** 10.1155/mi/3045323

**Published:** 2025-11-15

**Authors:** Yongjuan Zhao, Hualan Zhou, Ying Chen, Dianxuan Guo

**Affiliations:** Department of Geriatrics, The Affiliated Huaian Hospital of Xuzhou Medical University, The Second People's Hospital of Huai'an, Huaian 223002, China

**Keywords:** coronary chronic total occlusion, coronary collateral circulation, geriatric patients, multiple signaling axes

## Abstract

The multiple signaling axes might be concerned with the poor coronary collateral circulation (CCC). This research aimed to investigate the relationship between the poor CCC and multiple signaling axes (tumor necrosis factor-α [TNF-α]/interleukin-1β [IL1β]/IL8 pro-inflammatory cytokine signaling axis, toll-like receptor 4/myeloid differentiation factor 88/nuclear factor kappa-B [TLR4/MYD88/NF-κB] immune-inflammatory signaling axis, and transforming growth factor-β1/reactive oxygen species [TGF-β1/ROS] oxidative stress-inflammatory signaling axis) in geriatric patients with coronary chronic total occlusion (CCTO). We simultaneously assessed the expressions of multiple signaling axis markers (TNF-α, IL1β, IL8, TLR4, MYD88, NF-κB, TGF-β1, and ROS) in geriatric patients with CCTO. The CCC was scored as follows: Grade 0 (without contrast filling), Grade 1 (filling of collateral vessels with no epicardial filling), Grade 2 (partial filling of epicardial arteries), and Grade 3 (fully filling of the epicardial arteries). The CCC Grade 2 group in patients with CCTO showed decreased TNF-α, IL1β, IL8, TLR4, MYD88, NF-κB, TGF-β1, and ROS compared with CCC Grade 1 group (*p* < 0.002), and the CCC Grade 1 group had lower levels of these markers than CCC Grade 0 group (*p* < 0.002). In conclusion, our findings may support the causative roles for the pro-inflammatory cytokine signaling axis (TNF-α/IL1β/IL8), immune-inflammatory signaling axis (TLR4/MYD88/NF-κB), and oxidative stress-inflammatory signaling axis (TGF-β1/ROS) in impairing CCC and poor formation of CCC in geriatric CCTO patients.

## 1. Introduction

Coronary total occlusions are characterized as myocardial cell ischemia, hypoxia as well as necrosis contributing to the irreversible myocardial injury due to the nonrenewable of myocardial cells [1]. Coronary collateral circulation (CCC) supplies blood to the ischemic myocardium after coronary arterial occlusion. The CCC is related to the improved survival rate after coronary artery occlusion with myocardial infarction [2]. The CCC development is the important cardioprotective process during coronary chronic total occlusion (CCTO) [2]. When the coronary arteries are totally obstructed, the CCC will develop through the mechanism of artery formation preserving cardiac function, rescuing ischemic tissues and reducing the death risk among patients with CCTO [3]. Good CCC is the potential treatment for patients with CCTO who did not receive percutaneous coronary intervention [3]. Inflammation inhibits the collateral circulation establishment playing an important role in the CCC formation through contributing to oxidative stress response and coronary endothelial dysfunction [4].

The pro-inflammatory cytokine storm originates from the pro-inflammatory response. The myocardial infarction with ventricular decompensation, pro-inflammatory cytokine levels are increased greatly, which induces a pro-inflammatory cytokine storm worsening prognosis [5]. The excessive pro-inflammatory cytokine storm initiates multiple pro-inflammatory signaling pathways. The different expression levels of pro-inflammatory signaling pathways are associated with different grades of CCC formation [5]. The activated pro-inflammatory signaling pathways are associated with collateral vessel dilatation with thin walls leading to collateral vessel rupture and bleeding. Pro-inflammatory cytokines and pro-inflammatory signaling pathways interweave as well as interact with each other, forming an interconnected pro-inflammatory network, which inhibits the CCC formation [5].

Aging in older people is related to low levels of inflammation. Age-associated low-grade and sterile chronic inflammation is termed inflamm-aging—it is not only a consequence of the increasing chronological ages, but also is a biomarker of biological aging including cellular senescence and immunosenescence [6]. However, the limitation of previous researches is that, unlike conventional aging model animals in laboratories like rats as well as mice, literatures on the association between aging and CCC in patients are very limited [7–11]. There are no studies that assess how aging affects the CCC formation in humans [7–11].

The collateral-dependent flow recoveries, collateral numbers, and collateral diameters are decreased and collateral rarefactions are induced following acute artery obstruction in aging rats as well as cats [7–11]. Aging impairs collateral flow recovery and decreases collateral circulation in the brain and hindlimb of mice. Aging also reduces the vasodilation caused by the endothelial cell dysfunction in cerebral and hindlimb collateral vessels in mice and rats [7–11]. However, there are no researches in humans that have investigated the relationship between the aging and collateral remodeling [7–11]. The available data on the impacts of aging on collateral circulation in patients are very limited because it is difficult to measure the collaterals and collateral-dependent flow of patients. Therefore, the researches performed in the experimental animals contributing to these above conclusions had no parallel patient researches with which to compare [7–11]. Further studies evaluating CCC based on artery obstruction in large numbers of patients are needed to investigate the effects of aging on collaterals [7–11].

Serum tumor necrosis factor-α (TNF-α) leads to vascular injury. TNF-α induces inflammatory vascular damage and stimulates vascular fibrosis as well as proliferation of smooth muscle cells, subsequently causing artery stiffness with left ventricular hypertrophy [12]. TNF-α increases expression of inflammatory genes associated with vascular dysfunction. The inhibition of TNF-α inflammatory pathway might be beneficial for preventing vascular injury [12]. Interleukin-1β (IL1β) plays an important role in pro-inflammatory response. Increased IL1β is associated with pro-inflammatory response and inflammatory vessel damage. High levels of IL1β promotes vascular damage mediated by the microparticles. IL1β is the mechanism for systemic vascular damages [13]. IL8 levels are highly expressed through damaged arteries in different of diseases initiating a pro-inflammatory cascade at the location of vascular injury and pro-inflammatory cell infiltration. IL8 expression attenuates neointima formation post-endoluminal vascular injuries causing monocyte cells/macrophage cells and T cells recruitment to the damaged arteries [14]. Toll-like receptor 4 (TLR4) leads to many elderly-related cardiovascular diseases involving in cardiovascular damage. TLR4 triggers the intracellular inflammatory signaling pathways activating downstream pro-inflammatory response, contributing to the releases of pro-inflammatory factors. The TLR4 signaling pathway shows association between pro-inflammatory response and cardiac vascular damage [15]. TLR4 expression is increased in the myocardium and arteries promoting cardiovascular dysfunction, and TLR4 plays a key role in the cardiac vascular pathology [15]. Myeloid differentiation factor 88 (MYD88) signaling pathway is an intracellular adopter protein that triggers pro-inflammatory signaling cascade promoting inflammatory response [16] and plays a key role in ischemic damage and leads to immuno-pro-inflammatory response aggravating tissue and cellular injury. MYD88 increases the expressions of pro-inflammatory factors (IL-6 as well as TNF-α) via activations of different inflammatory signaling pathways [17]. The over-activation of the nuclear factor kappa-B (NF-κB) signaling pathway regulates the expressions of pro-inflammatory genes leading to the elevated levels of IL1β, IL-6, IL8 as well as TNF-α as inflammatory cytokines activating the pro-inflammatory responses [18]. The transforming growth factor-β1 (TGF-β1) is associated with the artery stiffness and vascular aging. Arteriosclerosis induced by TGF-β1 plays an important role in promoting vascular age and arterial stiffness which is an independent risk factor for artery stiffness [19]. TGF-β1 expression increases the excessive collagen deposition as well as accelerates the elastin degradation in the artery walls [19]. Excessive concentration of reactive oxygen species (ROS) leads to the imbalance of the generation and elimination of ROS and oxidative stress response causing vascular cell injury at the molecular as well as cellular level. Over-expression of ROS induces the adverse effects on the cardiovascular systems [20] promoting the pro-inflammatory cell participation, severe lipid peroxidation as well as adverse vessel remodeling. ROS/oxidative stress participate in the initiation and development of vessel atherosclerosis and vascular restenosis [21].

There are no relevant researches focusing on the roles of different signaling axes in CCC formation in geriatric CCTO patients. Due to the complexity of CCC formation, there are substantial difficulties to establish a clear relationship between clinical symptoms and impaired CCC. We hypothesized that only a single biomarker of vascular injury or signaling axis cannot clearly evaluate CCC after CCTO. Therefore, the present integrated researches of multiple signaling axes (TNF-α/IL1β/IL8 pro-inflammatory cytokine signaling axis, TLR4/MYD88/NF-κB immune-inflammatory signaling axis, and TGF-β1/ROS oxidative stress-inflammatory signaling axis) were try to better understand their roles in the formation of CCC and to understand the mechanism of poor formation of CCC during CCTO in geriatric patients.

## 2. Materials and Methods

### 2.1. Study Populations

This research enrolled 640 geriatric patients with CCTO lesions, and the inclusion criteria for selecting patients were as follows: (1) geriatric patients aged 65–87 years old and (2) patients with right coronary artery chronic total occlusion (RCA CTO), left circumflex artery CTO (LCX CTO), and left anterior descending artery CTO (LAD CTO). This research project was submitted as well as approved by The Affiliated Huaian Hospital's Human Research Ethics Review Committee (Number AHH-XMU-18-22) to ensure the research data are safety in line with the laws and regulations in China. All the subjects accepted to take part in the research through the forms of informed consent in accordance with the Declaration of Helsinki for medical study involving human participants. The criteria for exclusion were as follows: (a) acute coronary occlusions, (b) two-vessel coronary occlusions, (c) multiple-vessel coronary occlusions, (d) geriatric patients without coronary occlusions, and (e) patients with coronary artery bypass graft.

### 2.2. Definitions in This Research

① CCTO is defined as the completely occluded coronary artery without the antegrade blood flow as well as a duration of over 3 months [22]. ② Silent myocardial infarction is defined as the electrocardiographic evidences of myocardial infarction without the clinical confirmation of myocardial infarction [23]. ③ Refractory angina is defined as the chronic disease (3 more months) characterized by ischemic heart disease, which cannot be improved after medical therapies [24]. ④ Tachycardia is defined as heart rate >100 beats per minute [25]. ⑤ Refractory hypertension is defined as the blood pressure that is not controlled despite taking five antihypertensive drugs [26]. ⑥ Chronic pain is defined as the pain lasting and recurring for up to more than 3 months [27]. ⑦ Heart failure is defined as the clinical syndromes due to cardiac dysfunction that inlvolved other tissues/organs, leading to a complex multiple systemic diseases [28]. ⑧ Diabetes is defined as having hemoglobin A1c of ≥6.5% and fasting plasma glucose of >7.0 mmol/L [29].

### 2.3. The Protocol of Research

Healthy participants (243) who came to the physical exam center of our hospital were enrolled into control (CON) group. The CON subjects were age matched with research participants. We screened 213 diagnosed patients with RCA CTO, 215 cases with LCX CTO, and 212 cases with LAD CTO included in this research. All patients were randomly assigned into the Grade 0 group, Grade 1 group, Grade 2 group, and Grade 3 group based on CCC grades (Grade 0, without contrast filling; Grade 1, filling of collateral vessels with no epicardial filling; Grade 2, partial filling of epicardial arteries, and Grade 3, fully filling of the epicardial arteries).

In the present study, the changes in the myocardial activation of multiple signaling axes connecting CCC poor formation were investigated in CCTO geriatric patients. The serum activation markers (TNF-α/IL1β/IL8, TLR4/MYD88/NF-κB, and TGF-β1/ROS) of pro-inflammatory cytokine signaling axis, immune-inflammatory signaling axis, and oxidative stress-inflammatory signaling axis were determined by the enzyme-linked immunosorbent assay in geriatric CCTO patients with poor formation of CCC.

#### 2.3.1. Tissue-Level Measurements

The tissue-level characteristic parameters of cardiac magnetic resonance image were measured in geriatric patients with CCTO. The myocardial tissue-level characteristics as indicators for subtle diffuse myocardial fibrosis, edema, and dysfunction included the late gadolinium enhancements, myocardial tissue morphology, T1 mapping-derived extracellular volume fractions, native T2 values, myocardial function, cardiac thickness, myocardial relaxation time, ventricular global longitudinal peak systolic strain, as well as peak systolic strain values. The myocardial tissue was measured by three cardiac imaging experts who were blinded to the clinical data of CCTO patients.

### 2.4. Evaluating CCC by Coronary Angiography

Coronary angiographies were performed by two experienced interventional cardiologists, and the Rentrop classification was used for evaluating CCC formation. To standardize the definition of coronary collateral formation, all coronary angiograms were analyzed to determine the classification of CCC [30]. The interventional cardiologists were blinded to the clinical outcome in geriatric patients. For the analyses, geriatric patients were classified as Rentrop CCC Grade 0 (absence of collateral vessels), Rentrop CCC Grade 1 (presence of small collateral vessels), Rentrop CCC Grade 2 (partial filling of distal branches), and Rentrop CCC Grade 3 (complete coronary collaterals) [30].

### 2.5. Laboratory Analyses of Serum TNF-α/IL1β/IL8

The samples of blood for cytokine analyses were taken from the peripheral veins under conditions of fasting in the morning. Serum samples of all subjects were collected from clotted venous blood by the immediate centrifugations (2000 rpm for 15 min, 4°C), and they were stored at −70°C until cytokine assays [31]. TNF-α levels in serum were detected by the enzyme-linked immunoabsorbent assays (ELISAs) with commercially available test kits (human TNF-α ELISA kit from Invitrogen, Camarillo, California, USA). The detection range for TNF-α was 15.2–1000 pg/mL. The absorbance levels were measured at the wavelength of 450 nm, and the results obtained of the CON subjects and the patients were expressed as pg/mL [31]. The serum samples were used to measure concentrations of IL1β by human ELISA assay kits (R&D Systems, Inc., Minneapolis, Minnesota, USA) according to the kit manufacturer's protocols. All serum samples were evaluated in duplicate, and based on the ELISA kit information, the serum assay range and serum sensitivity were 5.9, 240, and 4.57 pg/mL, respectively [32]. The human IL8 ELISA kits (Invitrogen, Camarillo, California, USA) were used for detecting human IL8 levels. The specific antibodies for IL8 were precoated on to the microplates. Standards as well as samples were transferred into the 96-well microplates. The antibody specific for IL8 was pipetted into the micro-ELISA plate. The color intensity was measured at a wavelength of 450 nm via a microplate reader [33].

### 2.6. Laboratory Measurements of TLR4/MYD88/NF-κB

The samples of morning fasting venous blood were collected from all participants. Serum samples were separated by centrifugation (3000 rpm for 10 min at 4°C). The samples of serum were stored at −80°C until the test time. Double antibody-sandwich ELISA techniques (R&D Systems, Minneapolis, Minnesota, United States) were used for the analyses of the expression levels of serum TLR4 (detection range: 0.415–45 ng/mL; sensitivity: 0.164 ng/mL). The absorbance values were measured at 450 nm via the enzyme standardizers. TLR4 levels were calculated in according with the standard curves supplied by the ELISA kits [34]. The expression levels of circulating serum MYD88 were measured with human MYD88 ELISA kits supplied by Abcam corporation (Cambridge, Massachusetts, United States) following the MYD88 ELISA kit's instructions, and the limits of detections for the ELISA kits were 17.3–1000 ng/L [35]. The levels of NF-κB in blood serum were measured by human NF-κB ELISA technology (Biorbyt Ltd, Cambridge, United Kingdom), and the experimental measurements were performed according to the manufacturers' protocols. The detection ranges for human NF-κB kits were between 7.0 and 100 mg/mL. The assays of NF-κB concentrations had high sensitivity as well as specificity for detecting the human NF-κB [36].

### 2.7. Detections of the Levels of TGF-β1/ROS

The serum samples were collected from geriatric patients with CCTO and healthy controls. Serum samples were aliquoted as well as stored at −70°C until further analyses. Analyzed TGF-β1 associated with vascular injury was quantified by the ELISA kits in serum. The serum TGF-β1 levels were evaluated with the human TGF-β1 ELISA kits (R&D Systems, Inc., Minneapolis, Minnesota, USA). The limits of detections were 1.53 ng/mL for TGF-β1. All serum samples were determined in duplicate, and the TGF-β1 values were analyzed in patients [37]. Human ROS ELISA kits were used to measure the expression levels of ROS in serum. The measurements were performed strictly in accordance with the ROS ELISA test kit instructions (Sigma-Aldrich, St. Louis, Missouri, United States) [38]. The working solutions for ROS were prepared, and the ROS standard solutions were diluted to the standard concentrations. The samples were placed on the microplate readers (Bio-Rad, Hercules, California, USA), and the optical density values of the samples were measured at 450 nm [38].

### 2.8. Statistical Analyses

Data were analyzed using SPSS 24.0 (IBM SPSS Statistics, Armonk, New York, United States) and were displayed as the mean ± standard deviations. The levels of significance were assessed through the one-way analyses of variance (ANOVA) and post hoc test (Tukey) among the various groups. The *p* values of <0.05 were considered as indicative of statistical and clinical significance [39]. The numerical data were presented as numbers as well as percentages. The comparisons of categorical variables among different groups were measured by the chi-square tests [40]. The Shapiro–Wilk tests were performed to measure whether the distributions of the parameters were normal distributions [41]. Using the software G^*⁣*^*∗*^^Power 3.1.9.7, the sample sizes were calculated as 640 geriatric patients, with 243 subjects in the CON group [42].

### 2.9. Data Analysis on ELISA and Graphical Presentation

**Figure 1:**
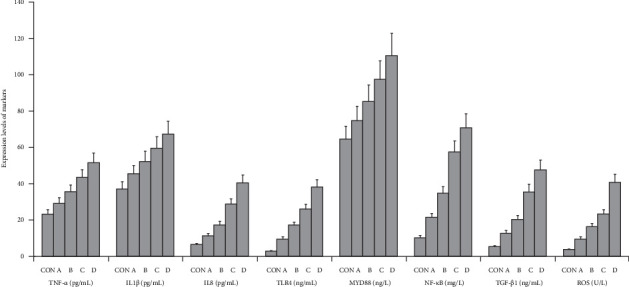
The results in graphical form for the ELISA data analysis in RCA CTO patients with different CCC grades. CON, control; A, CCC Grade 3; B, CCC Grade 2; C, CCC Grade 1; D, CCC Grade 0.

**Figure 2:**
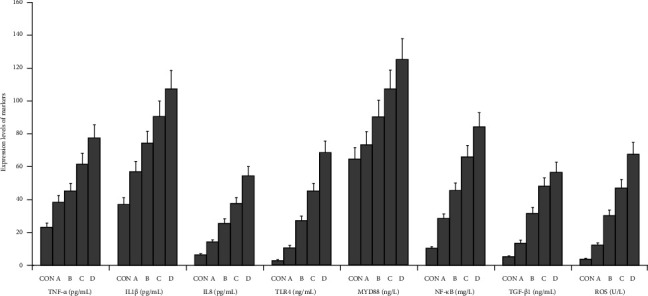
The graphical form for determining the concentration of markers associated with CCC in geriatric patients with LCX CTO. CON, control; A, CCC Grade 3; B, CCC Grade 2; C, CCC Grade 1; D, CCC Grade 0.

**Figure 3:**
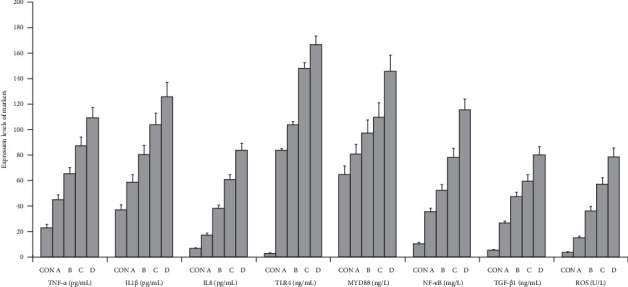
The ELISA data analyses in a graphical form in geriatric LAD CTO patients with CCC. CON, control; A, CCC Grade 3; B, CCC Grade 2; C, CCC Grade 1; D, CCC Grade 0.

**Figure 4:**
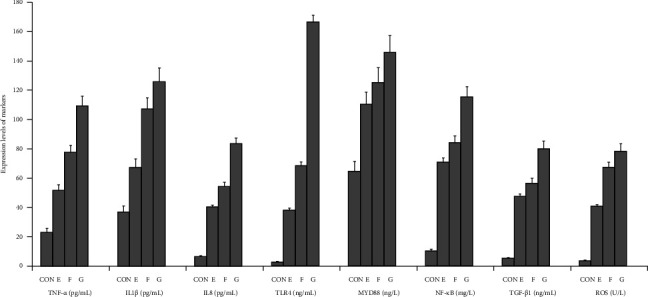
Graphical analysis of ELISA data shown. CON, control; E, CCC Grade 0 in RCA CTO patients; F, CCC Grade 0 in LCX CTO patients; G, CCC Grade 0 in LAD CTO patients.

The graphical methods were utilized for the ELISA data analysis in geriatric CCTO patients with different CCC formation (Figures 1–4).

### 2.10. Animal Experimental Studies

The mouse care and experimental protocols were approved by the Experimental Animal Care and Use Committee of The Affiliated Huaian Hospital of Xuzhou Medical University (HA 2017-54) and conducted in accordance with the Guide for the Care and Use of Laboratory Animals (the US National Institutes of Health). Kunming (KM) mice were used for in vivo research, which were purchased from the Xuzhou Medical University Animal Center (Xuzhou, China) with License Number SCXK (SU) 2017-0009. All KM mice received humane care were kept in a standard 12 h light–dark cycle at 22–24°C. Mice were acclimated for 1 week before the tests began. The myocardial infarction model in mice was induced by the permanent ligation of the left anterior descending coronary artery, and the sham operative control group was set as the CON. Coronary collaterals were measured using contrast echocardiography (mice myocardial blood flow). The male KM mice were randomized into the following four different groups (10 mice per group) according to the contrast echocardiography results: CON group (*n* = 10), young mice group with good collaterals (*n* = 10), adult mice group with less collaterals (*n* = 10), as well as aged mice group with impaired collaterals defined as lack of collaterals (*n* = 10). Serum samples were drawn from the inferior vena cava in the anesthetized mice to determine the concentrations of TNF-α, TLR4, NF-κB, and ROS. The samples were then centrifuged for 30 min at 840 × *g* at 4°C to separate the blood serum. Serum levels of TNF-α, TLR4, NF-κB, and ROS were evaluated using the mouse TNF-α, TLR4, NF-κB, and ROS ELISA Kits (Abcam, Cambridge, Massachusetts, USA) exactly according to the manufacturer's suggestions.

## 3. Results

### 3.1. Clinical Characteristics of Geriatric Patients With CCTO and CON Subjects

The clinical characteristics of geriatric patients and non-CCTO subjects were summarized in Table 1, which showed that there were no statistically significant differences among all groups in gender, age, silent myocardial infarction, refractory angina pectoris, tachycardia, refractory hypertension, chronic pain, hemoglobin A1c levels, heart failure, and diabetes.

### 3.2. Poorly/Well-Developed CCC in Different Coronary Artery CTO

The percentage of CCC formation was shown in Table 2. Meanwhile, 44% of LAD CTO patients had CCC Grade 0%, and 31% had CCC Grade 1; among LCX CTO patients, 35% had CCC Grade 0, and 25% had CCC Grade 1; among RCA CTO patients, 23% had CCC Grade 0, 1and 3% had CCC Grade 1, with significant difference among LAD CTO, LCX CTO, and RCA CTO patient groups (*p* < 0.05). Compared with RCA CTO and LCX CTO patient groups, LAD CTO patient group was more likely to have a poor CCC.

### 3.3. Correlation Between Biomarker Levels and CCC Formation in Geriatric Patients With RCA CTO

**Figure 5:**
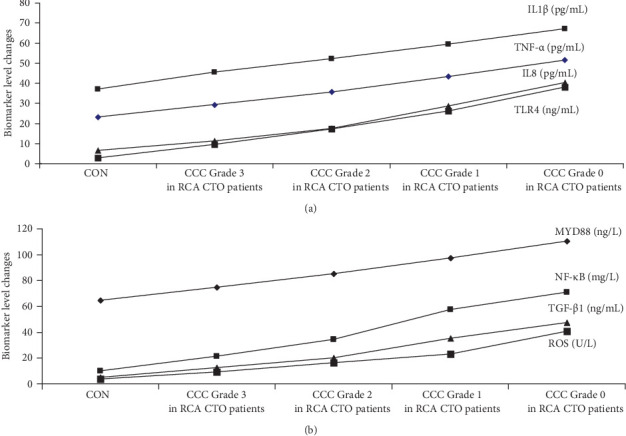
(a, b) Marker expression associated with CCC in geriatric patients with RCA CTO. *p* < 0.002 (CON group/CCC Grade 3 in RCA CTO patient group). *p* < 0.002 (CCC Grade 3 in RCA CTO patient group/CCC Grade 2 in RCA CTO patient group). *p* < 0.002 (CCC Grade 2 in RCA CTO patient group/CCC Grade 1 in RCA CTO patient group). *p* < 0.002 (CCC Grade 1 in RCA CTO patient group/CCC Grade 0 in RCA CTO patient group). Group comparisons (CON group/CCC Grade 3 in RCA CTO patient group/CCC Grade 2 in RCA CTO patient group/CCC Grade 1 in RCA CTO patient group/CCC Grade 0 in RCA CTO patient group) were made using ANOVA, *p* < 0.002. Adjustments for age, sex, silent myocardial infarction, refractory angina pectoris, tachycardia, refractory hypertension, chronic pain, hemoglobin A1c levels, heart failure, and diabetes. CCC, coronary collateral circulation; CON, control; CTO, chronic total occlusion; IL1β, interleukin-1β; IL8, interleukin-8; MYD88, myeloid differentiation factor 88; NF-κB, nuclear factor kappa-B; RCA, right coronary artery; ROS, reactive oxygen species; TGF-β1, transforming growth factor-β1; TLR4, toll-like receptor 4; TNF-α, tumor necrosis factor-α.

RCA CTO patients with CCC Grade 2 group had the higher TNF-α/IL1β/IL8, TLR4/MYD88/NF-κB, and TGF-β1/ROS levels compared with CON group and RCA CTO patients with CCC Grade 3 group (*p* < 0.002). The further analyses showed that RCA CTO patients with CCC Grade 0 exhibited higher expression of TNF-α/IL1β/IL8, TLR4/MYD88/NF-κB, and TGF-β1/ROS than the RCA CTO patients with CCC Grade 2 and RCA CTO patients with CCC Grade 1 (*p* < 0.002) (Figure 5a,b). These experiments demonstrated that the activation of signaling axes (TNF-α/IL1β/IL8, TLR4/MYD88/NF-κB, and TGF-β1/ROS) may promote poorly developed CCC and inhibit the good CCC formation in RCA CTO patients (Figure 5a,b).

### 3.4. Analyses of the Changes of Signaling Axis Activation in LCX CTO Patients

**Figure 6:**
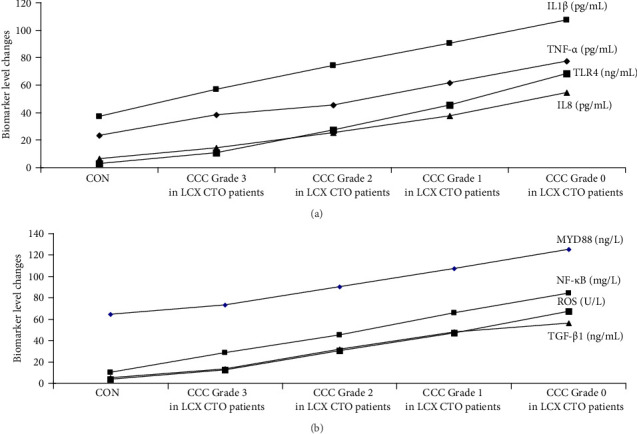
(a, b) Effects of marker levels on CCC formation in LCX CTO. *p* < 0.002 (CON group/CCC Grade 3 in LCX CTO patient group). *p* < 0.002 (CCC Grade 3 in LCX CTO patient group/CCC Grade 2 in LCX CTO patient group). *p* < 0.002 (CCC Grade 2 in LCX CTO patient group/CCC Grade 1 in LCX CTO patient group). *p* < 0.002 (CCC Grade 1 in LCX CTO patient group/CCC Grade 0 in LCX CTO patient group). Group comparisons (CON group/CCC Grade 3 in LCX CTO patient group/CCC Grade 2 in LCX CTO patient group/CCC Grade 1 in LCX CTO patient group/CCC Grade 0 in LCX CTO patient group) were made using ANOVA, *p* < 0.002. Adjustments for age, sex, silent myocardial infarction, refractory angina pectoris, tachycardia, refractory hypertension, chronic pain, hemoglobin A1c levels, heart failure, and diabetes. CCC, coronary collateral circulation; CON, control; CTO, chronic total occlusion; IL1β, interleukin-1β; IL8, interleukin-8; LCX, left circumflex coronary artery; MYD88, myeloid differentiation factor 88; NF-κB, nuclear factor kappa-B; ROS, reactive oxygen species; TGF-β1, transforming growth factor-β1; TLR4, toll-like receptor 4; TNF-α, tumor necrosis factor-α.

The activations of multiple signaling axes may be primarily responsible for the poor CCC formation. The TNF-α/IL1β/IL8, TLR4/MYD88/NF-κB, and TGF-β1/ROS of LCX CTO patients with CCC Grade 2 were significantly higher than that of CON subjects and patients with CCC Grade 3, respectively (*p* < 0.002). The levels of TNF-α/IL1β/IL8, TLR4/MYD88/NF-κB, and TGF-β1/ROS were significantly higher in patients with CCC Grade 0 than in patients with CCC Grades 1 and 2, respectively (*p* < 0.002; Figure 6a,b). These results indicated that the overactivation of multiple signaling axes may accelerate the poor CCC formation and restrict the good CCC development in geriatric LCX CTO patients, underscoring the roles of multiple signaling axes as CCC inhibitors potentially associated with poor CCC (Figure 6a,b).

### 3.5. Upregulation of Multiple Signaling Axes Associated With CCC Poor Formation in LAD CTO Patients

**Figure 7:**
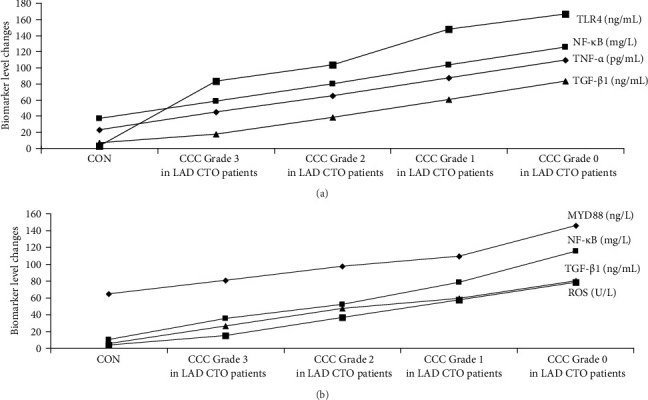
(a, b) Analyses of the circulating markers in geriatric LAD CTO patients with CCC. *p* < 0.002 (CON group/CCC Grade 3 in LAD CTO patient group). *p* < 0.002 (CCC Grade 3 in LAD CTO patient group/CCC Grade 2 in LAD CTO patient group). *p* < 0.002 (CCC Grade 2 in LAD CTO patient group/CCC Grade 1 in LAD CTO patient group). *p* < 0.002 (CCC Grade 1 in LAD CTO patient group/CCC Grade 0 in LAD CTO patient group). Group comparisons (CON group/CCC Grade 3 in LAD CTO patient group/CCC Grade 2 in LAD CTO patient group/CCC Grade 1 in LAD CTO patient group/CCC Grade 0 in LAD CTO patient group) were made using ANOVA, *p* < 0.002. Adjustments for age, sex, silent myocardial infarction, refractory angina pectoris, tachycardia, refractory hypertension, chronic pain, hemoglobin A1c levels, heart failure, and diabetes. CCC, coronary collateral circulation; CON, control; CTO, chronic total occlusion; IL1β, interleukin-1β; IL8, interleukin-8; LAD, left anterior descending coronary artery; MYD88, myeloid differentiation factor 88; NF-κB, nuclear factor kappa-B; ROS, reactive oxygen species; TGF-β1, transforming growth factor-β1; TLR4, toll-like receptor 4; TNF-α, tumor necrosis factor-α.

Compared with CON participants and LAD CTO patients with CCC Grade 3, LAD CTO patients with CCC Grade 2 showed an increased upregulation of TNF-α/IL1β/IL8, TLR4/MYD88/NF-κB, and TGF-β1/ROS (*p* < 0.002), and LAD CTO patients with CCC Grade 0 had higher levels of TNF-α/IL1β/IL8, TLR4/MYD88/NF-κB, and TGF-β1/ROS compared with LAD CTO patients with CCC Grades 1 and 2 (*p* < 0.002; Figure 7a,b). These data demonstrated that the activation and upregulation of TNF-α/IL1β/IL8, TLR4/MYD88/NF-κB, and TGF-β1/ROS played the significant roles in development of CCC in geriatric LAD CTO patients. The positive regulations of multiple signaling axes led to impaired coronary collateral vessel development (Figure 7a,b).

### 3.6. Comparing Activations of the Signaling Axes in Three Patient Groups With CCC Grade 0

**Figure 8:**
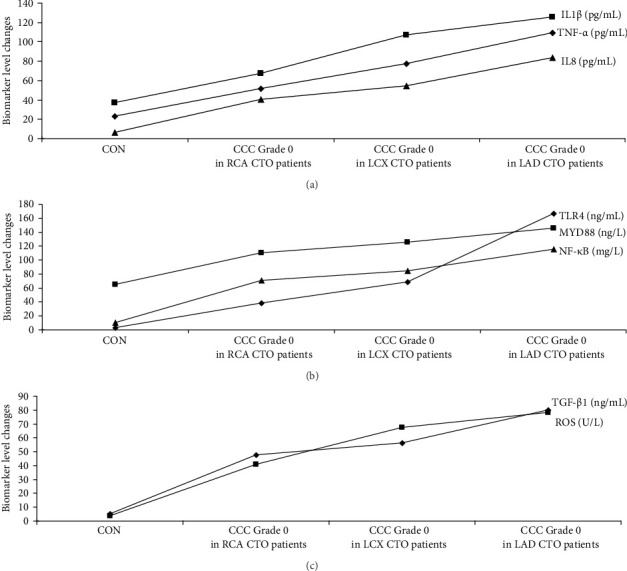
(a–c) Assessments of the CCC in geriatric patients with CCTO. *p* < 0.04 (CON group/CCC Grade 0 in RCA CTO patient group). *p* < 0.04 (CCC Grade 0 in RCA CTO patient group/CCC Grade 0 in LCX CTO patient group). *p* < 0.04 (CCC Grade 0 in LCX CTO patient group/CCC Grade 0 in LAD CTO patient group). Group comparisons (CON group/CCC Grade 0 in RCA CTO patient group/CCC Grade 0 in LCX CTO patient group/CCC Grade 0 in LAD CTO patient group) were made using ANOVA, *p* < 0.04. Adjustments for age, sex, silent myocardial infarction, refractory angina pectoris, tachycardia, refractory hypertension, chronic pain, hemoglobin A1c levels, heart failure, and diabetes. CCC, coronary collateral circulation; CON, control; CTO, chronic total occlusion; IL1β, interleukin-1β; IL8, interleukin-8; LAD, left anterior descending coronary artery; LCX, left circumflex coronary artery; MYD88, myeloid differentiation factor 88; NF-κB, nuclear factor kappa-B; RCA, right coronary artery; ROS, reactive oxygen species; TGF-β1, transforming growth factor-β1; TLR4, toll-like receptor 4; TNF-α, tumor necrosis factor-α.

The levels of activations of multiple signaling axes (TNF-α/IL1β/IL8, TLR4/MYD88/NF-κB, and TGF-β1/ROS) were significantly increased in the LAD CTO patients with CCC Grade 0 when compared with RCA CTO patients with CCC Grade 0 and LCX CTO patients with CCC Grade 0 (*p* < 0.04). The results showed that geriatric patients with LAD CTO displayed more serious impairment in CCC formation (Figure 8a–c).

### 3.7. Multiple Regression Analyses of Risk Factors Related to Poor CCC Formation in Geriatric Patients With CCTO

To analyze the independent risk factors linked to the poor CCC formation, the multiple regression analyses were performed to explore independent risk factors of poor CCC formation in geriatric CCTO patients. As shown, the three signaling axes (TNF-α/IL1β/IL8, TLR4/MYD88/NF-κB, and TGF-β1/ROS) were the independent risk factors for poorly formed CCC in geriatric patients with CCTO after adjustments for age, sex, silent myocardial infarction, refractory angina pectoris, tachycardia, refractory hypertension, chronic pain, hemoglobin A1c levels, heart failure, and diabetes (Table 3).

### 3.8. Influences of TNF-α, TLR4, NF-κB, and ROS on Coronary Collaterals in Mice Myocardial Infarction

**Figure 9:**
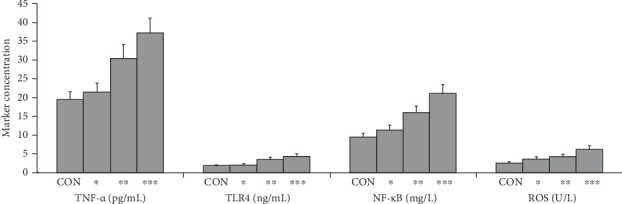
The levels of TNF-α, TLR4, NF-κB, and ROS of different age groups. CON, control; *⁣*^*∗*^, young mice group with good collaterals; *⁣*^*∗∗*^, adult mice group with less collaterals; *⁣*^*∗∗∗*^, elderly mice group with impaired collaterals. *p* < 0.05 (CON group/young mice group with good collaterals). *p* < 0.05 (young mice group with good collaterals/Adult mice group with less collaterals). *p* < 0.05 (adult mice group with less collaterals/elderly mice group with impaired collaterals). Group comparisons (CON group/young mice group with good collaterals/adult mice group with less collaterals/elderly mice group with impaired collaterals) were made using ANOVA, *p* < 0.05. NF-κB, nuclear factor kappa-B; ROS, reactive oxygen species; TLR4, toll-like receptor 4; TNF-α, tumor necrosis factor-α.

At 4 weeks after permanent coronary artery ligations, the levels of TNF-α, TLR4, NF-κB, and ROS were increased in adult mice group with less collaterals when compared with the CON group as well as young mice group with good collaterals (*p* < 0.05), and the concentrations of TNF-α, TLR4, NF-κB, and ROS were further elevated in aged mice group with impaired collaterals compared with the young mice group with good collaterals and the adult mice group with less collaterals (*p* < 0.05). These findings suggested that the elevated levels of TNF-α, TLR4, NF-κB, and ROS may inhibit the collateral formation in mice with myocardial infarction. These preliminary experimental data showed that the high levels of TNF-α, TLR4, NF-κB, and ROS might affect the formation of collaterals in myocardial infarction mice (Figure 9).

## 4. Discussion

The establishment of CCC is key to improving the prognosis of myocardial infarction. Pro-inflammatory cytokines regulate the activations of different signaling pathways forming the complex inflammatory network, which inhibits the information of collateral circulation [5]. The inflammatory cytokine storm signaling is related to the CCC development of postmyocardial infarction. The growth of CCC reduces mortality in patients with myocardial infarction and recurrent myocardial infarction [5]. The increased level of pro-inflammatory cytokine TNF-α impacts the angiogenesis of collateral circulation. The different levels of TNF-α expressions after myocardial infarction are related to the various grades of CCC information. TNF-α affects the short-term and long-term prognoses for myocardial infarction by inhibiting the generation of CCC [5]. The vascular inflammatory response and vessel endothelial dysfunction lead to ischemic injury [43]. The inflammatory IL1β signaling pathway promotes the injury of arterial endothelial cells by activating ROS stress response. The increase of IL1β is closely associated with the damage of vessel endothelial cells [44]. The IL8 as a biomarker of inflammatory response is highly expressed in the walls of the pathological vessels and strongly related to cardiovascular events playing a key role in cardiovascular illnesses [45]. The overexpressed IL8 is critical for the initiation as well as progression of arterial adverse remodeling [45].

In the present research, the overexpressions of TNF-α, IL1β, and IL8 associated with poor CCC formation were identified as well as validated by the analyses of pro-inflammatory cytokine signaling axis (TNF-α/IL1β/IL8), where geriatric CCTO patients with CCC poor formation expressed high levels of TNF-α, IL1β, and IL8. In the analyses of pro-inflammatory cytokine signaling axis, the differences in levels of TNF-α/IL1β/IL8 were clearly observed among CCC Grades 0, 1, 2, and 3 groups. Comparison with data from healthy controls also supported that the TNF-α/IL1β/IL8 signaling axis was selected as a marker for evaluating CCC poor formation. These results also showed that the LAD CTO was more likely to form poorer CCC than RCA CTO and LCX CTO. The association between LAD CTO and poor CCC formation may be due to the higher levels of expression of TNF-α/IL1β/IL8 signaling axis. Our results revealed that the TNF-α/IL1β/IL8 signaling axis constrained the arteriogenic capacity and could potentially inhibit CCC. The relationship of TNF-α/IL1β/IL8 signaling axis and CCC poor formation in our research may indicate a potential mechanism for poor CCC development, that is, the activation of TNF-α/IL1β/IL8 pro-inflammatory cytokine signaling axis may lead to vascular endothelial cell dysfunction [46], inhibition of pro-angiogenic factor expression [47], and downregulation of pro-angiogenic factor activity [48, 49].

The dysregulation of immune pro-inflammation leads to the vasculopathy with endothelial cell injury causing vascular endothelial cell dysfunction and arterial occlusion. The immune-inflammatory signaling plays the important roles in affecting the macro- and microcirculations [50]. TLR4 activates the immune response and promotes the releases of pro-inflammatory cytokines resulting in pro-inflammatory reaction [51]. The pro-inflammatory immune responses contribute to the vascular endothelial dysfunction (vascular hyperpermeability and hypercoagulability) impairing collateral circulation as well as organ function [51]. MYD88 is expressed in the vascular endothelial cells. MYD88 signaling alters the vascular endothelial cell function as well as contributes to the pathological process of vascular diseases [52]. MYD88 activity promotes the activation of NF-κB signaling pathway and induces the transcription of inflammatory cytokines, resulting in the pro-inflammatory responses. MYD88 signaling leads to the vascular inflammatory response related to the pro-inflammatory immune responses [52]. The activated NF-κB-mediated vascular endothelial inflammation aggravates pro-inflammatory vascular response, blood vessel permeability, and vascular endothelial cell injury. NF-κB signaling pathway induces the endothelial cellular dysfunction [53]. The drugs targeting the NF-κB signaling pathway may prevent inflammatory vascular response-induced endothelial damage [53].

In this research, we focused primarily on the TLR4/MYD88/NF-κB regulatory effects and mechanisms of immune-inflammatory signaling axis in the poor CCC formation of geriatric CCTO patients. The main findings were as follows: (1) Activated immune-inflammatory signaling axis could induce the poor CCC formation evidenced by the overexpressions of TLR4/MYD88/NF-κB in geriatric CCTO patients; (2) increased immune-inflammatory signaling could play the key roles in reducing pro-angiogenic signaling [54] in vascular endothelial cells and inhibiting the formation of good collateral circulation in ischemic myocardium; (3) the activated immune-inflammatory signaling axis (TLR4/MYD88/NF-κB) was independently related to the increased risks of poor developed CCC in geriatric CCTO patients. We found that TLR4/MYD88/NF-κB immune-inflammatory signaling axis could influence arterial physiology leading to arterial stiffening and vascular dysfunction related to impaired angiogenesis [55, 56], suggesting that the TLR4/MYD88/NF-κB immune-inflammatory signaling axis may serve as the coactivator of both immune and pro-inflammatory responses.

The oxidative stress-inflammation axis plays an important role in the pathogenesis of diseases [57]. The oxidative stress leads to the onset as well as progression of vascular dysfunction by impairing vasomotor regulation and adverse vascular remodeling [58]. The vascular oxidative stress occurring in the vasculature contributes to cells and tissues injury having the key roles in vascular endothelial dysfunction of the arterial vascular walls [59]. Increased oxidative stress promotes vasoconstriction and vascular stiffness playing an important role in the initiation as well as progression of vascular damage and vascular diseases [59]. The inflammatory response of vascular walls increases the dysfunction of vascular endothelial cells. The vicious circle of oxidative stress and pro-inflammatory response leads to the progression of atherosclerosis [60]. The TGF-β1 promotes an increase of the oxidative stress [61] and pro-inflammatory signaling [62]. TGF-β1 is induced by oxidized low-density lipoprotein as well as contributes to the vascular endothelial cell injury associated atherosclerosis [63]. Oxidative stress is caused by the ROS overexpression and plays an important role in vascular endothelial cell death in different ways [64]. The excessive ROS leads to cellular injury and also activates the redox transcription factor NF-kB, contributing to pro-inflammatory cytokine expression in the vessel walls [60].

Based on the analyses of TGF-β1/ROS signaling, our findings were the following: (1) the geriatric CCTO patients with poor CCC had higher TGF-β1/ROS levels than patients with good CCC. (2) TGF-β1/ROS was independently related to CCC poor formation. (3) The inclusion of TGF-β1/ROS in this research increased the ability to determine the risk factors of poor CCC formation. (4) TGF-β1/ROS signaling markers might be the potentially reliable predictors for the early identification of poor CCC development in geriatric CCTO patients. Our further analyses found that the overexpression of TGF-β1/ROS oxidative stress-inflammatory signaling axis causing oxidative stress-inflammatory response had adverse impacts on the formation of good CCC leading to vascular endothelial injury. Under oxidative stress and pro-inflammatory conditions, the TGF-β1/ROS oxidative stress-inflammatory signaling axis contributed to the cardiac pericyte depletion as well as dysfunction inhibiting cardiac vascular formation [65]. We found that the activation of TGF-β1/ROS oxidative stress-inflammatory signaling axis may be the independent risk factor for the elevated risk of poor CCC in geriatric patients with CCTO and demonstrated a new mechanism by which high levels of TGF-β1/ROS oxidative stress-inflammatory signaling axis induced the poor collateral circulation via oxidative stress-inflammation response in geriatric patients with CCTO. Measurement of TGF-β1/ROS inflammatory signaling axis may be utilized for the early risk stratification of geriatric patients with CCTO, helping distinguish high-risk geriatric patients with CCC poor formation.

In summary, our research confirmed that the geriatric patients with CCTO had CCC poor formation and revealed the activation of multisignaling axes, which potentially promoted the CCC poor formation. The poor CCC occurrence is a complex process associated with multiple interconnected signaling axes, including pro-inflammatory cytokine signaling axis (TNF-α/IL1β/IL8), immune-inflammatory signaling axis (TLR4/MYD88/NF-κB), and oxidative stress-inflammatory signaling axis (TGF-β1/ROS).

The CCC formation is a complex process involving angiogenesis as well as arteriogenesis. Pro-inflammatory response and immune response play the key roles in the CCC formation. Pro-inflammatory response can inhibit the formation of the collateral circulation [5]. Pro-inflammatory response and immune response are the independent risk factors for the poor CCC formation. The endothelial progenitor cells play the important roles in angiogenesis and arteriogenesis, and pro-inflammatory response and immune response further affect the formation of CCC by increasing the oxidative stress response and contributing to endothelial cell dysfunction [5]. Moreover, oxidative stress promotes the activation of immuno-pro-inflammatory signaling pathways, which directly causes injury to the vascular endothelial progenitor cells leading to the poor CCC formation [5]. Our findings suggested that three signaling axes identified may be associated with the CCC poor formation. The three signaling axes (TNF-α/IL1β/IL8, TLR4/MYD88/NF-κB, and TGF-β1/ROS) played the inhibitory roles in CCC formation. These results suggested that the aberrantly high activations of three signaling axes (TNF-α/IL1β/IL8 pro-inflammatory cytokine signaling axis, TLR4/MYD88/NF-κB immune-inflammatory signaling axis, and TGF-β1/ROS oxidative stress-inflammatory signaling axis) might promote the poor CCC formation (Rentrop Grades 0–1) in geriatric CCTO patients.

Coronary heart disease mostly affects older people, showing an eight-fold increase in myocardial infarction incidences in the older age groups compared with people younger than 55 years of age [66]. Atherosclerotic plaque hemorrhage and pultaceous debris in atheromatous plaques are more common in the elderly. Elderly patients with coronary heart disease frequently have severe multivessel coronary artery occlusive disease with higher lesion complexity [66] inducing the excessive pro-inflammatory cytokines secreted postmyocardial infarction as well as the pro-inflammatory pathways activated by these pro-inflammatory cytokines are involved in the development of coronary lesions [67].

The monocytes and macrophages in the artery walls are interacted with modified low-density lipoproteins activating pro-inflammatory signaling pathways by inducing pro-inflammatory cytokine gene expression promoting the formation of foam cells and arterial plaques [68]. The activation of the pro-inflammatory pathways is the main response of the immunocytes to pro-atherogenic modified low-density lipoproteins resulting in upregulation of signaling pathways that involve the pro-inflammation-immune responses [68].

Our research further showed that the inflammatory-oxidative stress signaling axis was activated and promoted the poor CCC formation pathogenesis in geriatric patients with CCTO. Oxidative stress is considered the key pathogenic factor and upregulates pro-inflammatory pathways [69]. The releases of pro-inflammatory cytokines are able to promote the oxidative stress via the activation of specific cell enzyme and, in turn, oxidative stress can activate pro-inflammatory signaling pathways to further cause the OxInflammation vicious cycle [69]. OxInflammation is a consequence of the mutual activation between pro-inflammatory response and permanent oxidative stress in atherosclerosis [69].

The CCC formation is a complex process involving angiogenesis as well as arteriogenesis. Pro-inflammatory response and immune response play the key roles in the CCC formation. Pro-inflammatory response can inhibit the formation of the collateral circulation [70]. Pro-inflammatory response and immune response are the independent risk factors for the poor CCC formation. The endothelial progenitor cells play the important roles in angiogenesis and arteriogenesis, and pro-inflammatory response and immune response further affect the formation of CCC by increasing the oxidative stress response and contributing to endothelial cell dysfunction [70]. Moreover, oxidative stress promotes the activation of immuno-pro-inflammatory signaling pathways, which directly causes injury to the vascular endothelial progenitor cells leading to the poor CCC formation [70].

The occlusion of LAD is related to the highest risks of adverse clinical events and poor outcomes because of the large area of myocardial tissue supplied by the LAD when compared with RCA and LCX [71]. The infarcted myocardial area is larger in patients with LAD occlusion associated with increasing incidence of heart failure. The patients in the LAD occlusion have a higher risk of cerebral stroke owing to left ventricle mural thrombus [71]. Therefore, the potential pathophysiological reasons for higher marker levels of inflammatory response in the LAD CTO group compared with RCA CTO and LCX CTO groups may be that LAD occlusion causes more severe myocardial damage [72] when compared with RCA occlusion and LCX occlusion and triggers more intense pro-inflammatory response.

Our research had some limitations. The data quality can obviously impact the result accuracy. A significant limitation of using cross-sectional design is that serum markers reflect only systemic levels, the absence of data on functional cardiac outcomes including right ventricular, left ventricular, and biventricular functions with echocardiography and cardiac magnetic resonance imaging. The CCC formation mainly depended on subjective judgment of interventional cardiologists, and the Rentrop classification only showed the anatomical structure of collateral vessels, and it is impossible to evaluate the structure and function of the collateral blood vessels. We did not research the CCC in young as well as middle-aged patients with CCTO and whether it was associated with poor CCC formation.

The limited number of geriatric CCTO patients with different CCC grades might have biased the results. The larger sample sizes can enhance the analysis of results. Therefore, the researches with the large sample sizes should be performed to increase the reliability of the results. In this research, the different CCC grades were assessed using coronary artery angiography alone, without the uses of coronary intravascular ultrasonography. This was also the single-center research, and further large sample and multicenter researches are needed to confirm these results.

## 5. Conclusions

The findings of this research showed that (1) in comparison to CON subjects, CCTO patients had higher marker levels of pro-inflammatory-immune-oxidative stress axis; (2) at multiple regression analyses, levels of TNF-α/IL1β/IL8, TLR4/MYD88/NF-κB, and TGF-β1/ROS were significantly predictive of poor formation of CCC; (3) multiple regression analyses showed that high-serum TNF-α/IL1β/IL8, TLR4/MYD88/NF-κB, and TGF-β1/ROS were independent risk factors for poor formation of CCC in geriatric patients with CCTO; (4) particularly, geriatric LAD CTO patients with poor CCC formation had higher levels of pro-inflammatory-immune-oxidative stress in comparison to RCA CTO and LCX CTO patients, which warrant further detailed investigation in future studies to better understand the molecular mechanisms.

We gained the novel insights into the inhibitory roles of three signaling axes (TNF-α/IL1β/IL8, TLR4/MYD88/NF-κB, and TGF-β1/ROS) in CCC formation promoting the poor CCC formation. The three signaling axes identified in our research may be associated with the CCC poor formation. These results suggested that the aberrantly high activations of three signaling axes (TNF-α/IL1β/IL8 pro-inflammatory cytokine signaling axis, TLR4/MYD88/NF-κB immune-inflammatory signaling axis, and TGF-β1/ROS oxidative stress-inflammatory signaling axis) might be independent high-risk factors and potential predictors for the poor CCC formation in geriatric CCTO patients.

## Figures and Tables

**Table 1:** Description of the baseline characteristics of geriatric CCTO patients with CCC.

Variables	CON (*n* = 243)	RCA CTO patients with different CCC grades (*n* = 213)	LCX CTO patients with different CCC grades (*n* = 215)	LAD CTO patients with different CCC grades (*n* = 212)	*p* values^a^
Gender					
Male, *n* (%)	125 (51)	109 (52)	106 (49)	102 (48)	0.98
Female, *n* (%)	118 (49)	104 (48)	109 (51)	110 (52)	0.97
Age (years)	73.2 ± 5.3	75.4 ± 4.2	82.5 ± 5.4	85.4 ± 7.3	0.74
Silent myocardial infarction, *n* (%)	0	21 (10)	19 (9)	30 (14)	0.53
Refractory angina pectoris, *n* (%)	0	45 (21)	41 (19)	47 (22)	0.89
Tachycardia, *n* (%)	21 (8)	31 (13)	35 (16)	31 (14)	0.43
Refractory hypertension, *n* (%)	18 (7)	29 (14)	32 (15)	34 (16)	0.28
Chronic pain, *n* (%)	24 (10)	31 (15)	27 (13)	33 (16)	0.67
Hemoglobin A1c levels 7%–9%, *n* (%)	31 (13)	40 (19)	49 (23)	44 (21)	0.40
Heart failure, *n* (%)	0	44 (21)	40 (19)	48 (23)	0.82
Diabetes, *n* (%)	60 (25)	74 (35)	79 (37)	72 (34)	0.46

Abbreviations: CCC, coronary collateral circulation; CON, control; CTO, chronic total occlusion; LAD, left anterior descending coronary artery; LCX, left circumflex coronary artery; RCA, right coronary artery.

^a^Significance via chi-square test.

**Table 2:** CCC formation in geriatric CCTO patients.

Variables	RCA CTO patients with different CCC grades (*n* = 213)	LCX CTO patients with different CCC grades (*n* = 215)	LAD CTO patients with different CCC grades (*n* = 212)	*p* values^a^
CCC grade 0, *n* (%)	49 (23)	76 (35)	94 (44)	0.03
CCC grade 1, *n* (%)	28 (13)	54 (25)	66 (31)	0.02
CCC grade 2, *n* (%)	79 (37)	50 (23)	31 (15)	0.01
CCC grade 3, *n* (%)	57 (27)	35 (16)	21 (10)	0.01

Abbreviations: CCC, coronary collateral circulation; CTO, chronic total occlusion; LAD, left anterior descending coronary artery; LCX, left circumflex coronary artery; RCA, right coronary artery.

^a^Significance via chi-square test.

**Table 3:** Multivariate regression analyses of the independent risk factors related to poor CCC in geriatric CCTO patient.

Variables	Odds ratio	95% CI	*p* value
Gender	1.24	0.54–11.13	0.26
Age	4.37	0.68–12.13	0.31
Silent myocardial infarction	3.75	0.59–14.03	0.28
Refractory angina pectoris	1.29	0.67–1.32	0.15
Tachycardia	4.21	0.53–14.65	0.35
Refractory hypertension	3.46	0.79–7.21	0.62
Chronic pain	1.37	0.59–15.31	0.32
Hemoglobin A1c levels	4.31	0.81–12.06	0.29
Heart failure	2.40	0.69–3.98	0.42
Diabetes	1.39	0.67–1.42	0.17
TNF-α	6.39	1.56–10.13	0.01
IL1β	4.25	1.49–7.42	0.02
IL8	5.61	1.61–15.24	0.001
TLR4	3.41	1.50–11.03	0.01
MYD88	4.36	1.45–8.13	0.02
NF-κB	6.18	1.53–17.31	0.001
TGF-β1	3.90	1.41–8.01	0.01
ROS	6.24	1.57–16.20	0.001

Abbreviations: CCC, coronary collateral circulation; IL1β, interleukin-1β; IL8, interleukin-8; MYD88, myeloid differentiation factor 88; NF-κB, nuclear factor kappa-B; ROS, reactive oxygen species; TGF-β1, transforming growth factor-β1; TLR4, toll-like receptor 4; TNF-α, tumor necrosis factor-α.

## Data Availability

The data are available upon reasonable request from the corresponding author. No additional data and/or materials are available.
